# Supplementary material for: Population analyses reveal heterogenous encoding in the medial prefrontal cortex during naturalistic foraging
Source: eLife. 2026 Jan 5;13:RP93994. doi: 10.7554/eLife.93994 (PMC12768408; doi:10.7554/eLife.93994)
Supplement: Supplementary file 1. — Statistical test results for Figure 6C, showing avoidance/escape withdrawal decoding accuracy measured across varying dataset time windows. [file elife-93994-supp1.docx]

**Supplementary File 1. AW/EW prediction accuracy as a function of dataset time window.**

| Dataset time window | Statistical result | Significance |
| --- | --- | --- |
| −31 – −29 | *t*(39) = .24, *p* > .999 | NS |
| −11 – −9 | *t*(39) = 1.62, *p* = .701 | NS |
| −7 – −5 | *t*(39) = 5.381, *p* < .001 | *** |
| −6 – −4 | *t*(39) = 5.328, *p* < .001 | *** |
| −5 – −3 | *t*(39) = 4.610, *p* < .001 | *** |
| −4 – −2 | *t*(39) = 4.631, *p* < .001 | *** |
| −3 – −1 | *t*(39) = 4.544, *p* = .008 | ** |
| −2 – 0 | *t*(39) = 4.544, *p* < .001 | *** |
| −1 – +1 | *t*(39) = 8.505, *p* < .001 | *** |
